# Exploring the possibilities and limitations of customized large language model to support and improve cervical cancer screening

**DOI:** 10.1186/s12911-025-03088-3

**Published:** 2025-07-01

**Authors:** Viola Angyal, Ádám Bertalan, Péter Domján, Elek Dinya

**Affiliations:** 1 Semmelweis University Doctoral College, Health Sciences Division, Institute of Digital Health Sciences, Budapest, Hungary; 2 Semmelweis University, Doctoral College, Health Sciences Division Interdisciplinary Applied Health Sciences Program, Budapest, Hungary

**Keywords:** Custom GPT, Artificial intelligence, Cervical cancer, Prevention, Prompt engineering, Natural language processing

## Abstract

**Background:**

The rapid advancement of artificial intelligence, driven by Generative Pre-trained Transformers (GPT), has transformed natural language processing. Prompt engineering plays a key role in guiding model outputs effectively. Our primary objective was to explore the possibilities and limitations of a custom GPT, developed via prompt engineering, as a patient education tool, which delivers publicly available information through a user-friendly design that facilitates more effective access to cervical cancer screening knowledge.

**Method:**

The system was developed using the OpenAI GPT-4 model and Python programming language, with the interface built on Streamlit for cloud-based accessibility and testing. It initially presented questions to testers for preliminary assessment. For cervical cancer-related information, we referenced medical guidelines. Iterative testing optimized the prompts for quality and relevance; techniques like context provision, question chaining, and prompt-based constraints were used. Human-in-the-loop and two independent medical doctor evaluations were employed. Additionally, system performance metrics were measured.

**Result:**

The web application was tested 115 times over a three-week period in 2024, with 87 female (76%) and 28 male (24%) participants. A total of 112 users completed the user experience questionnaire. Statistical analysis showed a significant association between age and perceived personalization (p = 0.047) and between gender and system customization (p = 0.037). Younger participants reported higher engagement, though not significantly. Females valued guidance on screening schedules and early detection, while males highlighted the usefulness of information regarding HPV vaccination and its role in preventing HPV-related cancers. Independent evaluations by medical doctors demonstrated consistent assessments of the system’s responses in terms of accuracy, clarity, and usefulness.

**Discussion:**

While the system demonstrates potential to enhance public health awareness and promote preventive behaviors, encouraging individuals to seek information on cervical cancer screening and HPV vaccination, its conversational capabilities remain constrained by the inherent limitations of current language model technology.

**Conclusions:**

Although custom GPTs can not substitute a healthcare consultations, these tools can streamline workflows, expedite information access, and support personalized care. Further research should focus on conducting well-designed randomized controlled trials to establish definitive conclusions regarding its impact and reliability.

**Clinical trial number:**

Not applicable.

**Supplementary Information:**

The online version contains supplementary material available at 10.1186/s12911-025-03088-3.

## Background

The rapid advancement of artificial intelligence (AI) has led to significant breakthroughs in natural language processing (NLP), with transformer-based models such as Generative Pre-trained Transformers (GPT) at the forefront [1–3]. GPTs have evolved from generalized language models designed for various tasks into more specialized models known as custom GPTs. These models are tailored to meet specific needs across various domains, including healthcare and other industries. The proliferation of custom GPTs is transforming how organizations harness AI, enabling them to leverage the power of large language models (LLMs) while fine-tuning them to address their unique challenges and data requirements [4–6]. Custom GPTs, distinguished from their generalized counterparts by their domain-specific training and deployment, offer several advantages [7–11]. They can improve task performance by reducing irrelevant information processing, enhancing accuracy, and delivering outputs more aligned with specific contexts or industry norms, such as in healthcare prevention [12–14].

Prompt engineering techniques are one option for creating custom GPT models to disseminate information on specific topics effectively. It represents a less resource-intensive method of customizing GPTs, focusing on refining input prompts to guide the model’s responses without further training [15, 16]. This approach leverages the existing capabilities of a general LLM by carefully crafting the prompts that are fed into the model, enabling it to deliver domain-relevant outputs with minimal computational overhead [17]. In this method, domain-specific knowledge was encoded into the prompts themselves. Through iterative testing, the prompts can be optimized to improve the quality and relevance of the responses. Techniques such as context provision, question chaining, and prompt-based constraints can be employed to guide the model’s output toward desired outcomes [18]. Zero-shot and few-shot learning methods are essential in prompt engineering for LLMs, enabling task performance without the need for specific training data, particularly when labeled data is scarce. Zero-shot learning guides the model using a single prompt without examples, optimized through techniques like explicit instructions or structured templates. Approaches such as “chain-of-thought” prompting enhance model accuracy by promoting step-by-step reasoning in zero-shot scenarios. In contrast, few-shot learning incorporates a small number of task-specific examples within the prompt, helping the model recognize patterns and improve responses for complex tasks [19]. Coherence, relevance, and domain-specific accuracy can be used to compare the results across different prompts. A human-in-the-loop (HILT) evaluation process can also be conducted to assess the quality of the system’s responses, as well as different questionnaires can be used to evaluate user experience [20–25].

Once a system is developed, the custom GPT can be integrated into a cloud-based interface, such as Streamlit, to provide users with seamless access to its capabilities. Streamlit’s cloud-based platform allows for easy deployment, enabling real-time interaction and scalability, while facilitating a user-friendly experience for accessing tailored information generated by the custom GPT. Streamlit is an open-source Python framework designed to simplify the development and deployment of machine learning models and data-driven applications. It allows developers to create intuitive and interactive web applications with minimal coding effort. When integrated with a cloud-based infrastructure, Streamlit enables real-time access to custom GPT models, allowing users to interact with the model via a web browser without needing specialized software. Streamlit supports a wide range of features, including dynamic widgets, interactive charts, and real-time updates, which enhance user experience.

Cervical cancer screening can be categorized as secondary prevention, and this is one of the earliest recommended screening tests, which is recommended by the American Cancer Society and many other guidelines, including the WHO recommendation [26–28]. By enabling early detection of precancerous changes and timely intervention, it significantly reduces the incidence and mortality associated with cervical cancer [29]. This screening test saves lives and is recognized as one of the most essential and widely implemented preventive healthcare measures globally [30–35]. Despite the proven effectiveness of cervical cancer screening in reducing incidence and mortality, significant disparities persist across different countries and population groups. Low- and middle-income countries (LMICs) face major challenges due to limited healthcare infrastructure, while even high-income countries struggle with inequities in screening access among marginalized communities, immigrants, and individuals with low socioeconomic status. Language barriers, financial constraints, mistrust in healthcare systems, and inadequate referral pathways contribute to lower screening uptake and delayed diagnoses, ultimately reducing the effectiveness of early detection efforts. Cultural beliefs, stigma, and geographic barriers further exacerbate these disparities, preventing many individuals from accessing timely screening and follow-up care. To bridge this gap, innovative multi-faceted approaches are needed, including the integration of digital health solutions. Custom GPT-based conversational agents can enhance personalized awareness and patient education, addressing both informational and emotional barriers. These AI-driven tools can provide women with accessible information on screening opportunities and risk factors while also educating men on human papillomavirus (HPV) transmission and the benefits of vaccination. HPV vaccination plays a crucial role in cervical cancer prevention by protecting against the high-risk strains of HPV that are responsible for the majority of cervical cancer cases, significantly reducing the incidence of this disease [36–38]. By improving health literacy and engagement, custom GPTs have the potential to increase screening participation and contribute to more equitable cervical cancer prevention efforts. A further advantage of cervical cancer screening tests is their applicability to younger populations, who are generally more receptive to and engaged with digital healthcare solutions, such as internet-based platforms and AI-driven language models [39–41]. This screening test is recommended to begin at the age of 30, while certain guidelines suggest initiation even earlier, from 25 years old [42]. This demographic’s familiarity with technology may enhance the uptake and acceptance of such innovations in healthcare. Based on these advantages, we selected the topic of cervical cancer prevention to develop a custom GPT model aimed at improving patient education and preventive measures. The goal was to enhance the dissemination of information, guiding patients on what actions they should take or consider in relation to cervical cancer screening and prevention strategies. Even though this tool cannot replace consultations with healthcare professionals, it could serve as a complement to traditional prevention processes [43, 44].

This paper explores the possibilities and limitations of creating a custom GPT, developed via prompt engineering, as a patient education tool, which delivers publicly available information through a user-friendly design that facilitates more effective access to cervical cancer screening knowledge. The objective of this study was to investigate whether a customized GPT-based model could enhance access to relevant cervical cancer prevention information by delivering personalized advice tailored to individual user needs. Our hypothesis posited that a custom GPT model enhances the accessibility, comprehensibility, and adherence to cervical cancer guidelines, thereby improving patient education and assisting patients in accessing specific information. The system was designed to generate empathetic and supportive responses, fostering adherence to cervical cancer screening protocols and promoting awareness of HPV vaccination and prevention strategies. A HILT evaluation was employed to capture an overview of patient needs and to establish a clear direction for future development. Additionally, two independent medical doctors evaluated the system’s responses for frequently asked questions.

## Methods

### System architecture and components

#### Initial question flow

**Fig. 1:**
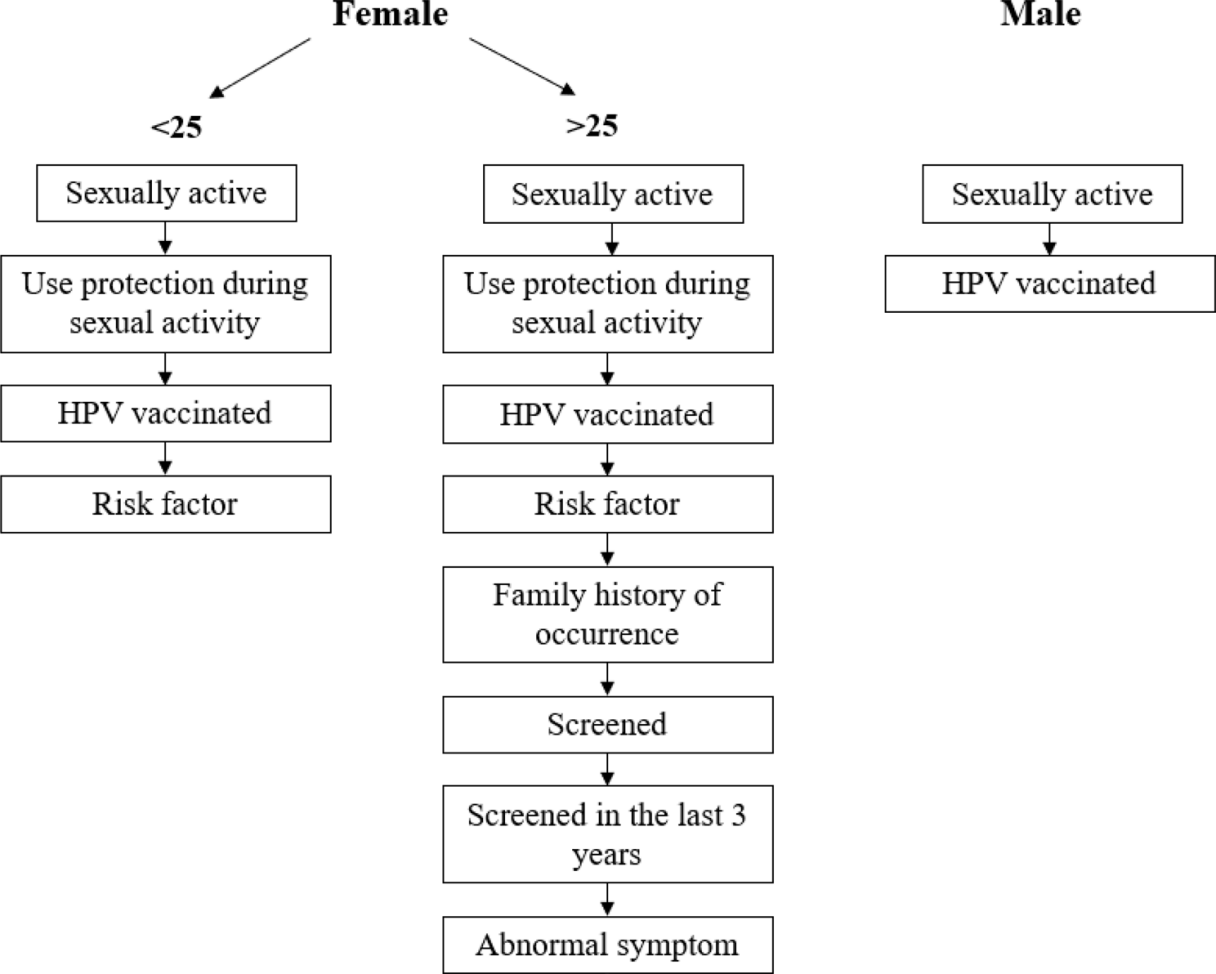
Question flow

Medical guidelines and recommendations related to cervical cancer, including HPV vaccination and prevention, were gathered to develop prompts and a question flow for our web application. The guidelines processed included those from the American Cancer Society and WHO for cervical cancer screening and treatment of pre-cancerous lesions, as well as relevant scientific publications. Additionally, two Hungarian guidelines—the Hungarian Ministry of Health’s Professional Protocol and recommendations from the Hungarian National Public Health and Pharmaceutical Center—were incorporated, as the system was tested in Hungary [26–28, 45–49]. Drawing from the literature and the feedback received during the drafting process, we developed a question flow that enables the system to present prompted questions for an anonymous preliminary assessment. The final parameters, assessed through questions, are shown below (Fig. 1). The survey questions initially asked by the system were developed specifically for our own custom GPT (Supplementary 1). Participants were informed about the purpose of the research and the use of their responses via informed consent. The completion of the questionnaire, which was anonymous and voluntary, implied their consent. Respondents were assured of their confidentiality, and participation was restricted to individuals who were at least 18 years old.

The system was designed in accordance with data minimization principles. It did not store any data, tailored responses based on the patient’s gender and age, dividing users into three groups: males (Male), females under 25 (Female < 25), and females over 25 (Female > 25), as per Hungarian screening guidelines. This allowed the system to provide personalized, context-specific information, along with emotional and informational support, encouraging users to ask further questions and take appropriate next steps in cervical cancer prevention.

#### Integration of OpenAI’s GPT-4 API

**Fig. 2:**
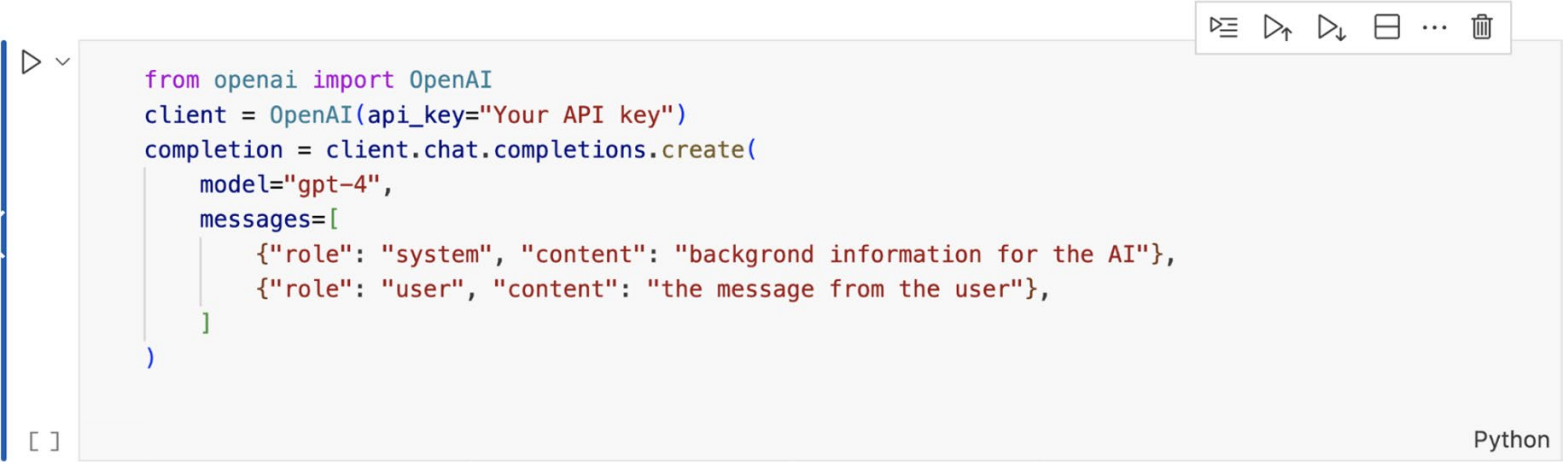
OpenAI GPT-4 model API integration into the system

The system was developed using OpenAI’s (San Francisco, CA, USA) GPT-4 model and Python programming language. We utilized OpenAI’s Application Programming Interface (API) for the GPT integration [50]. On the backend, the OpenAI API facilitated communication with OpenAI’s language models, enabling the system to process user inputs and generate appropriate responses. To integrate OpenAI’s API into the web application, an API key was obtained from the OpenAI API dashboard following user registration. Depending on the technology stack, the appropriate OpenAI library must be installed in the application’s Python environment. Since Python was used in our system, the integration was implemented using the OpenAI Python library, installed via *pip install openai*. To interact with OpenAI’s models, such as GPT-4, we used code in which our API key is assigned to the OpenAI object’s *api_key* argument (Fig. 2).

#### User interface

The user interface (UI) was developed using Streamlit, providing an interactive platform where users can input queries and receive responses efficiently. Streamlit is specifically designed for building web-based user interfaces using only Python code. To leverage its functionality, the streamlit library must be installed and imported into the application’s Python environment (*import streamlit as st*.). We opted for its cloud-based deployment feature, ensuring broad accessibility and sufficient computational resources for the application. This approach enabled remote user access and large-scale usability testing.

#### Prompt engineering

**Fig. 3:**
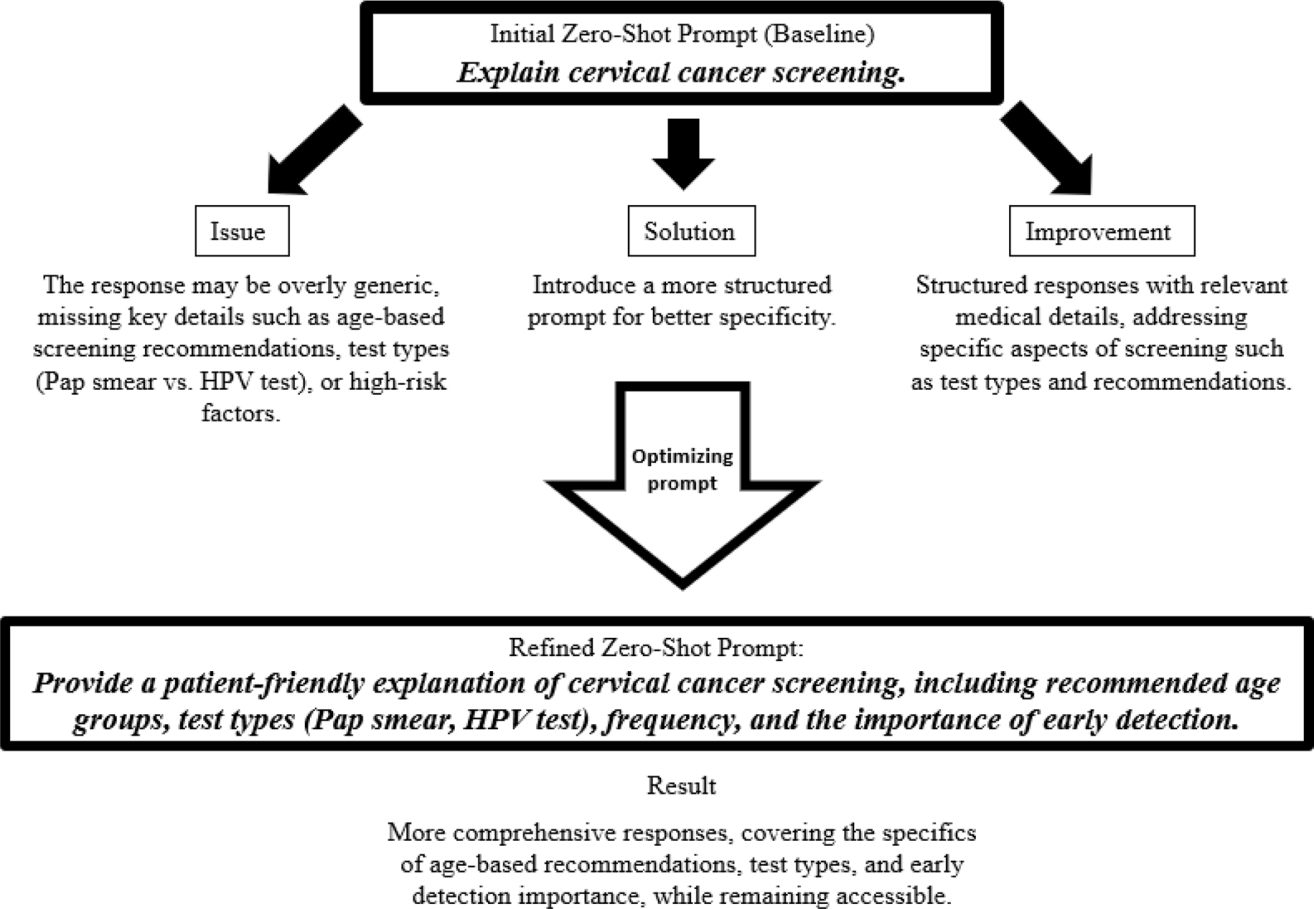
Optimizing prompts with zero-shot technique

During the prompt engineering process, we developed text-based instructions to optimize the LLM for generating more accurate and relevant outputs concerning cervical cancer prevention, and after every iteration smaller group of the developers (3–5 people) tested the outputs. The prompts were designed to provide structured guidance, regulating and refining the model’s responses. This approach ensures that the LLM remains contextually aligned with the intended informational goals, enhancing the precision and reliability of the generated content. Domain-specific prompts were refined iteratively to optimize the model’s accuracy and relevance. System, instructive, and contextual prompts were applied, with context provision used to embed cervical cancer screening information directly into the prompts. During the initial phase, the LLM’s task was defined via an initial prompt, a predefined instruction embedded in the code to guide response generation. This prompt establishes the context and domain, enabling the model to produce relevant outputs. The prompt given was: „ You are a healthcare assistant providing information on cervical cancer screening”. We employed the zero-shot learning mode, which involves using simple instructions as prompts without providing any examples (Fig. 3). This approach effectively elicits the expected responses from the LLM. The methodology aims to leverage the cognitive patterns developed by the LLM during its pre-training phase, enhancing its ability to generalize and produce accurate outputs in novel contexts. By utilizing zero-shot techniques, we optimized the model’s performance across various tasks, particularly in scenarios where labeled data is limited, thereby maximizing the model’s utility in diverse applications.

**Fig. 4:**
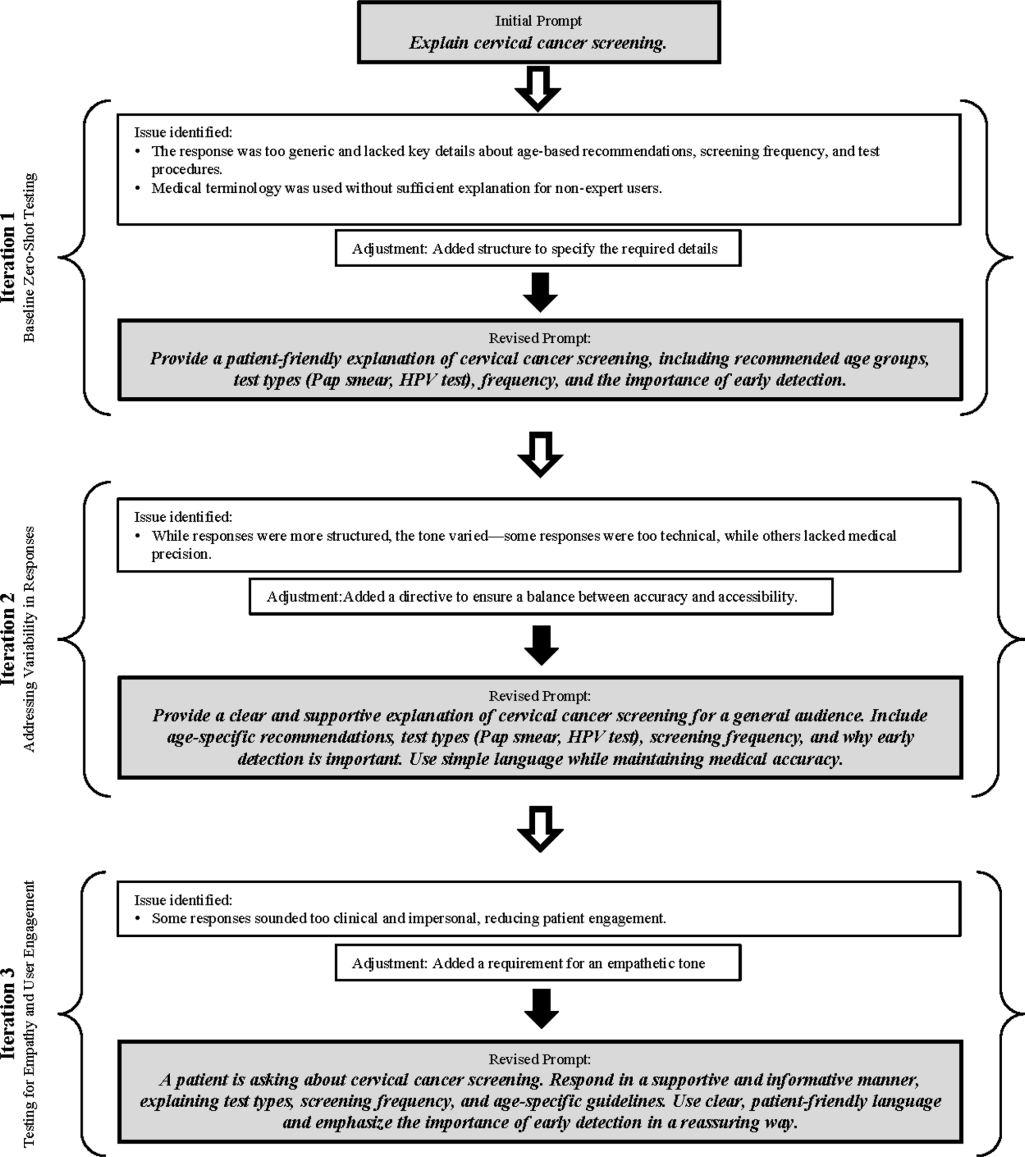
Example for the iteration process

The number of iterations performed in prompt optimization depends on the complexity of the task, user feedback, and the initial performance of the model. In the case of the AI-based custom GPT for the cervical cancer screening web application, the optimization process involved approximately 3–7 iterations in case of each prompt before reaching an optimal prompt structure (Fig. 4).

Question chaining guided the model through a logical series of queries, covering screening guidelines and prevention strategies, while prompt-based constraints ensured a focus on relevant content. Each iteration was evaluated for coherence and adherence, with continuous adjustments made to improve performance. To facilitate transparency, both the publicly available link to the GitHub repository for the system and the full list of applied prompts are included in Supplementary 2.

#### Visual elements

Additionally, external resources, including image assets, were incorporated to enhance user engagement. Visual elements such as banners and logos were utilized to improve the interface’s accessibility and usability.

### User experience questionnaire

Participants tested the web application by interacting with the custom GPT model for cervical cancer screening. Afterward, they completed an anonymous online survey with 10 questions. The questionnaire was specifically designed for the web application developed in this study. During the development phase, we reviewed established user experience questionnaires, such as the Chatbot Usability Questionnaire (CUQ) [20] and the User Experience Questionnaire (UEQ), to inform its design. However, to obtain more detailed data relevant to the specific requirements of our web application and support future development, the questions were tailored accordingly. A pre-test was conducted with a group of 4 researchers from the study team to evaluate the clarity, relevance, and effectiveness of the questionnaire. Feedback from this initial assessment was used to refine the questions and ensure they accurately captured user experience and usability aspects specific to the web application. A four-point scaled response format was implemented to systematically capture user feedback. For all questions, testers were able to select from four predefined answers, which included options ranging from the most positive to the most negative. To simplify the evaluation process, we used a numbered scale: 1 (totally agree), 2 (mostly agree), 3 (barely agree), and 4 (do not agree). This scale provided a clear visual representation, making the results easier to interpret and understand. To enhance the representativeness of the evaluation data in the tables, we categorized testers into three distinct subgroups based on age and gender. The first subgroup consists of female testers under the age of 25 (Female < 25). The second subgroup includes female testers aged 25 and above (Female > 25), while the third subgroup comprises all male testers (Male). This stratification allows for a more nuanced analysis of responses across demographic variables. We gathered feedback during 3 weeks in April 2024. The HITL evaluation collected feedback on relevance, customization, usability, and accuracy.

Descriptive statistics were used to characterize the system tester population. Continuous variables, such as age, were summarized using the mean and standard deviation, while testers were subsequently categorized into age groups, with percentage distributions calculated to establish a comprehensive demographic profile.

To assess the relationship between age groups and user experience parameters, the Chi-Square test was employed. This test was selected because both age group classifications and questionnaire responses are categorical variables. It is well-suited for determining whether the observed frequency distributions differ significantly from those expected under the null hypothesis (p < 0.05), thereby directly addressing age-related variations in user experience.

Furthermore, to evaluate potential differences in user experience parameters concerning gender, the non-parametric Mann–Whitney U test was applied. This test is appropriate for comparing two independent groups, such as male and female, especially when the data do not conform to a normal distribution or when the response variables are ordinal.

### Independent evaluation of the system’s responses by medical doctors

To further evaluate our custom GPT in responding to cervical cancer-related inquiries, we conducted an independent evaluation by two medical doctors. Thirty of the most frequently asked questions on cervical cancer screening were selected from the websites of the Hungarian National Public Health Center, PennMedicine, and the Mayo Clinic (Supplementary 3). The system was asked to provide answers to these questions, and both the questions and the generated responses were distributed to two independent medical doctors for evaluation. The evaluators were instructed to review each question along with the corresponding response and to rate the system’s answer based on three dimensions:

Accuracy: Does the answer align with current medical knowledge and practice guidelines?

Clarity: Is the answer easily understandable and logically structured?

Usefulness: Does the answer offer practical information or value for clinical use or patient education?

For each question, the evaluators provided a separate score for each dimension on a scale from 1 (Strongly Disagree/Not Accurate), 2 (Disagree), 3 (Agree) and 4 (Strongly Agree/Very Accurate). Additionally, the evaluators were invited to include brief written comments to elaborate on their ratings if necessary. To assess the independent evaluations of the system’s responses by two medical doctors (MD1 and MD2), we employed descriptive statistics and the Wilcoxon Signed Ranks Test.

## Results

### Characteristic of the system tester population

**Fig. 5:**
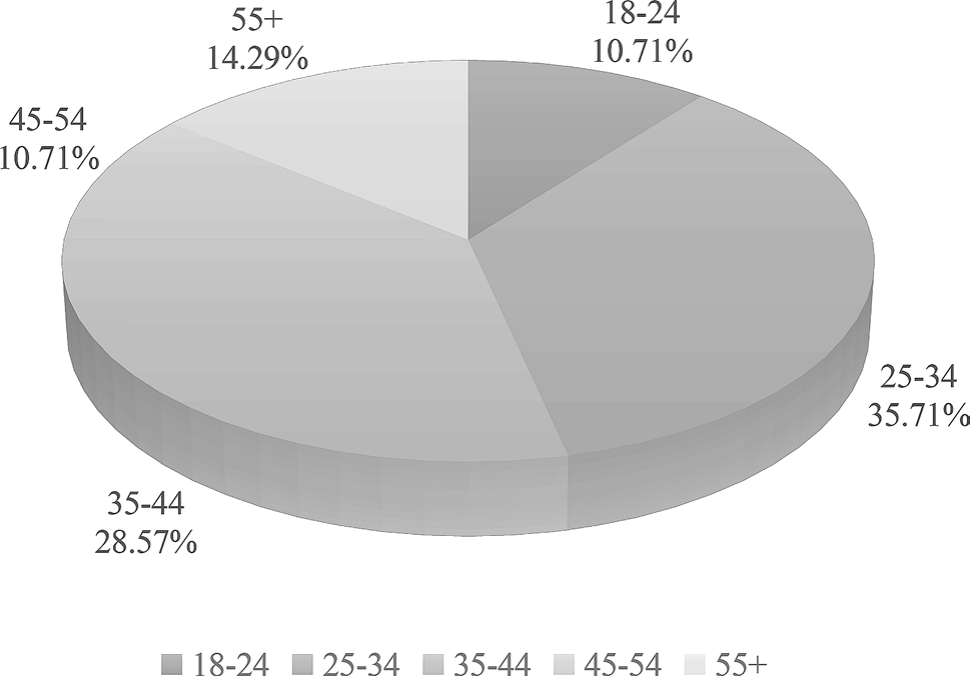
Age distribution of system tester population (Male)

**Fig. 6:**
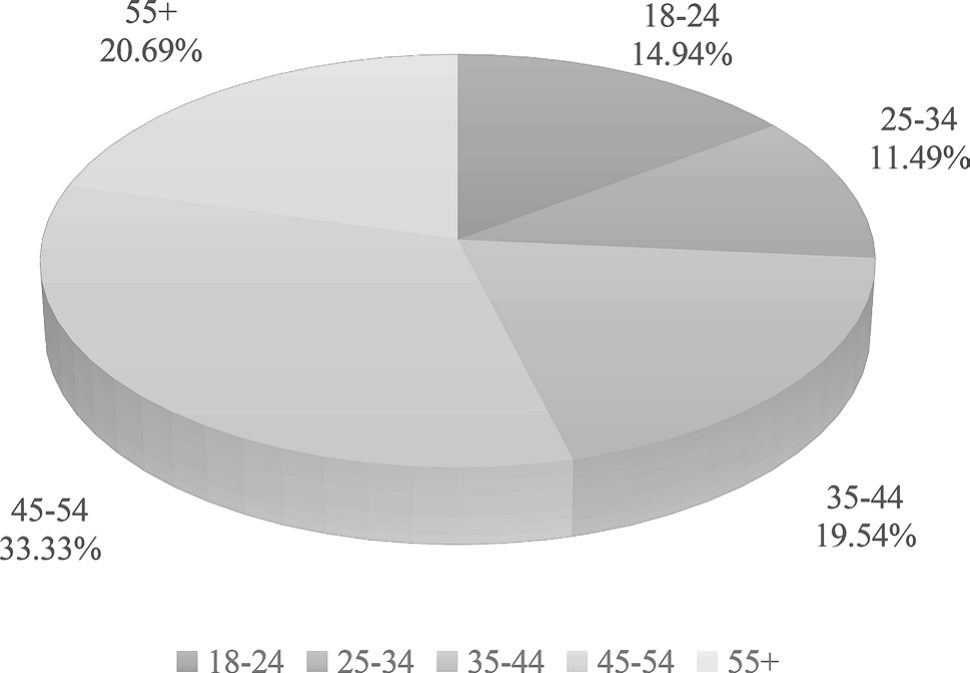
Age distribution of system tester population (Female)

Data collection was carried out over a three-week period during which the application was tested by a total of 115 testers (87 female (76%), 28 male (24%). The mean age was 41.2 (±SD 13.5), ranging from 18 to 74 years, and the majority of testers were female. The age distribution of the two biological genders is presented in Figs. 5 and 6. The age distribution among 87 female participants revealed that 16 (18%) were under the age of 25, while 71 (82%) were 25 years or older. Among all participants, we identified three subgroups. The first subgroup consisted of female testers under 25 years old (Female < 25), the second subgroup included female testers over 25 years old (Female > 25), and the third subgroup comprised male testers (Male).

In each subgroup, the majority of testers reported being sexually active, yet only 46.15% in the Female < 25 subgroup and 50% in the Female > 25 subgroup reported using protection during sexual activity (Table 1). These findings highlight potential risk factors for sexually transmitted infections, including HPV, which is directly linked to cervical cancer risk.

Based on participant responses, only 10.81% of females over 25 and 10.71% of males reported being HPV vaccinated. These findings highlight the need for targeted efforts to improve vaccination rates, particularly among males, who are often less engaged in HPV prevention strategies. In contrast, 69.23% of females under 25 years old reported being vaccinated, likely due to government financial support, as HPV vaccination has been provided free of charge since 2014 for 12-year-old children in 7th grade in Hungary. The introduction of new government financial support has led to a notable increase in HPV vaccination rates among young women, as reflected in the responses from our application testers.

Regarding risk factors and screening history among female participants, 46.15% reported at least one recognized risk factor for cervical cancer in the Female < 25 subgroup, and 21.62% in the Female > 25 subgroup, such as smoking. Importantly, 97.3% of females aged 25 and older reported having undergone cervical cancer screening at least once in their lives, with 77.03% having had a screening within the past three years. These screening rates reflect adherence to current cervical cancer screening guidelines, although gaps remain in reaching the full population. The Female > 25 subgroup was asked whether they had a family history of cervical cancer and if they had experienced any abnormal symptoms recently. Only 27.03% reported being aware of a family history of occurrence, and 10.81% indicated experiencing abnormal symptoms.

**Table 1:** Characteristic of the system tester population

	***n***		Parameters	TRUE		FALSE	
				***n***	***%***	***n***	***%***
Female < 25	13	Sexually active		11	84.62%	2	15.38%
		Use protection during sexual activity		6	46.15%	5	38.46%
		Risk factor		6	46.15%	7	53.85%
		HPV vaccinated		9	69.23%	4	30.77%
Female > 25	74	Sexually active		60	81.08%	14	18.92%
		Use protection during sexual activity		37	50.00%	23	31.08%
		Risk factor		16	21.62%	58	78.38%
		HPV vaccinated		8	10.81%	66	89.19%
		Family history of occurrence		20	27.03%	54	72.97%
		Screened		72	97.30%	2	2.70%
		Screened within the last 3 years		57	77.03%	17	22.97%
		Experience abnormal symptoms		8	10.81%	66	89.19%
Male	28	Sexually active		24	85.71%	4	14.29%
		HPV vaccinated		3	10.71%	25	89.29%

### Performance metrics

**Fig. 7:**
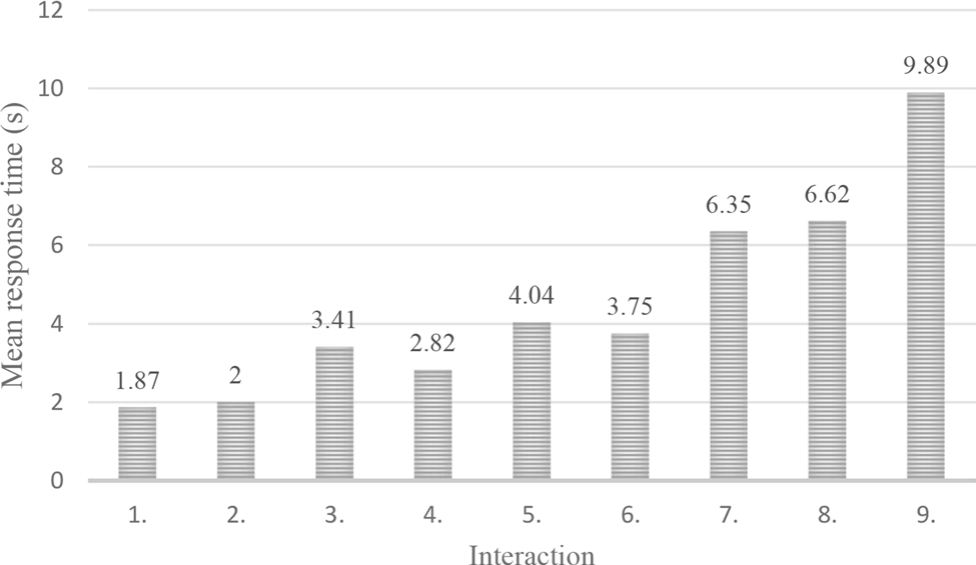
System response time

Response time was measured using the Python module (datetime.now), based on 11 tests. In these tests, we measured the interval between receiving OpenAI’s response and its display on the Streamlit interface. It is important to note that our system was deployed via a Streamlit cloud-based service, meaning that speed metrics are influenced by internet speed and browser performance as well in addition to the underlying model. In general, OpenAI’s API responded within 500 milliseconds to 3 seconds; however, this range varied with factors such as query complexity, network bandwidth, and the user’s browser environment. Our results indicate that the mean response time increased with each interaction, likely due to the system reprocessing the entire context from the beginning (Fig. 7). No API failures, timeouts, or user input errors were observed during testing. Regarding scalability, leveraging Streamlit and OpenAI’s API inherently supports flexible scaling, provided that API rate limits and cloud resources are effectively managed.

### Results of the user experience questionnaire

Among the 115 web application testers, 112 participants completed the feedback form, providing data critical to evaluating the application’s performance and overall user experience (Table 2). Initial impressions were largely positive, with 81.25% of participants indicating a positive experience (“mostly agree”), and 15.18% expressing a highly favorable impression (“totally agree”). In contrast, 3.57% reported a more negative first impression (“barely agree”). These findings underscore the importance of enhancing positive user engagement while identifying factors that may lead to negative initial responses.

In terms of system customization, 55.36% of participants mostly agreed, and 33.93% barely agreed, that the application was tailored to individual needs. The user interface was regarded as entirely intuitive by 51.79% of testers, who felt it was clear without requiring pretraining or guidance, while an additional 38.39% mostly agreed it was accessible. However, 8.93% only barely agreed with it, and 0.89% did not find the interface straightforward. These findings suggest a need for continued optimization in system customization to better meet individual requirements while ensuring the interface remains user-friendly and accessible. Most participants found the system easy to navigate, with 40.18% totally agreeing and 54.45% mostly agreeing that it was simple to use; 4.46% only barely agreed, and 0.89% did not find it user-friendly. These findings emphasize the importance of maintaining ease of navigation to support a seamless user experience for diverse users. Feedback on interactivity revealed that 12.5% of respondents totally agreed the system was engaging, 70.54% mostly agreed, 16.07% barely agreed, and 0.89% disagreed. These findings highlight the value of increasing the interactive elements of the system to enhance user engagement and satisfaction.

The relevance of system-generated responses was rated highly, with 22.32% describing responses as entirely relevant and 74.11% as mostly relevant; only 3.57% barely agreed with this, and none found responses irrelevant. These findings underscore the need to maintain a high standard for response relevance to ensure the system meets user’s expectations for meaningful interaction. Participants also assessed personalization, with 12.4% totally agreeing that the system’s answers were customized to their specific queries, while 62.5% mostly agreed, 13.39% barely agreed, and 11.62% felt the responses were too general. On adaptability to user needs, 22.32% indicated the system completely adjusted to individual requirements, 63.39% mostly agreed, 11.61% barely agreed, and 2.68% found it unresponsive to personal needs. These findings highlight the need to further refine the system’s capacity for generating personalized content while maintaining its usability to ensure broad accessibility and user satisfaction.

Participants were also asked whether they would use the system in the future and if they would recommend it to others. Only 6.25% would certainly use it again, while 36.61% mostly agreed, 41.96% barely agreed, and 15.18% stated they would not return to the system. In terms of recommendations, 2.54% would definitely suggest the application to others, with 63.39% mostly agreeing, 12.5% barely agreeing, and 3.57% not endorsing it. These findings indicate the importance of improving features that could enhance long-term usability and encourage positive user recommendations.

**Table 2:** User experience questionnaire responses

	1		2		3		4	
	totally agree		mostly agree		barely agree		do not agree	
	*n*	*%*	*n*	*%*	*n*	*%*	*n*	*%*
Positive first impression	17	15.18%	91	81.25%	4	3.57%	0	0.00%
Customization of the system	9	8.04%	62	55.36%	38	33.93%	3	2.68%
Clarity of the interface usability	58	51.79%	43	38.39%	10	8.93%	1	0.89%
Simplicity of system usage	45	40.18%	61	54.46%	5	4.46%	1	0.89%
System interactivity level	14	12.50%	79	70.54%	18	16.07%	1	0.89%
Relevance of responses	25	22.32%	83	74.11%	4	3.57%	0	0.00%
Personalization of advice	14	12.50%	70	62.50%	15	13.39%	13	11.61%
Adaptability to personal needs	25	22.32%	71	63.39%	13	11.61%	3	2.68%
Future reuse	7	6.25%	41	36.61%	47	41.96%	17	15.18%
Recommend to others	23	20.54%	71	63.39%	14	12.50%	4	3.57%

**Fig. 8:**
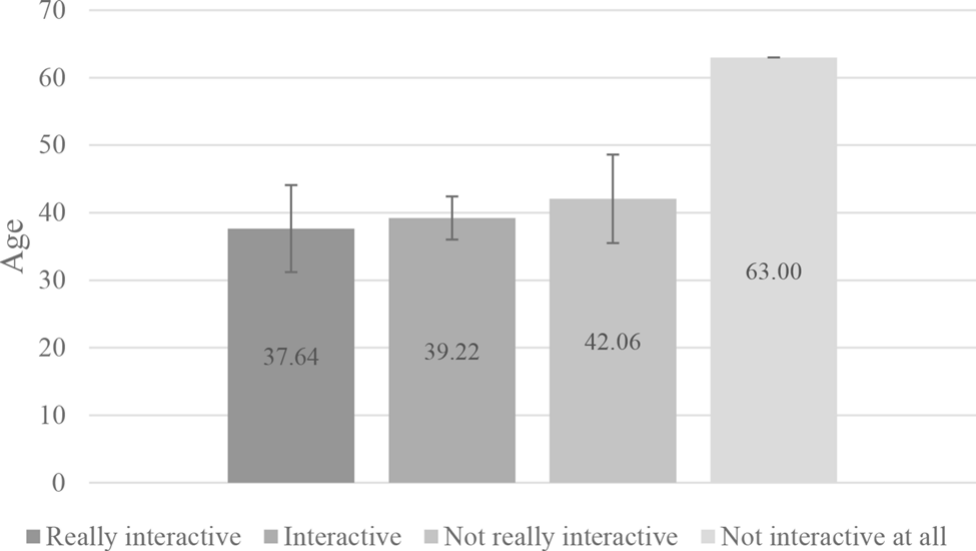
Web application interactivity perceived by different age groups with 95% confidence interval

Analyzing further the data, we noticed that younger participants, mean age of under 40 years, generally reported a higher level of engagement with the interface, describing it as more interactive and user-friendly compared to testers over 40 years (Fig. 8). This trend may be attributed to younger individuals’ greater familiarity with digital platforms and their comfort in navigating AI-driven tools. In contrast, older users, while still able to use the application, often perceived it as less intuitive. The overall user participants mean age, who completed the user experience questionnaire, was 39.69 years old. The tester’s mean age, who found the user interface really interactive, was 37.64, and interactive was 39.22. The tester’s mean age was 42.06, who found the application not too interactive, and 63 years old, who found it not interactive at all. However, testing the results with the Chi-Square Test, the relationship between age and system interactivity was not significant in the different age groups (p = 0.91). This might highlight the need for user interface adaptations to ensure accessibility and ease of use across different age groups, ensuring that healthcare tools such as this custom large language model are effective for a broad demographic. However, it is also notable that not only can age influence how interactive a person finds a user interface, but other sociological factors may also have an impact, such as location, education level, local infrastructure, income, etc., which were not measured by our user experience questionnaire.

Regarding the parameters assessed in the user experience questionnaire, we identified a statistically significant association with the Chi-Square Test (p < 0.05) between age groups and the perceived personalization of the responses, with a p-value of 0.047 (Table 3). However, no significant associations were found for the other 9 variables assessed. Additionally, concerning gender and the examined variables, we observed a significant association (p = 0.037) with Mann–Whitney U test between gender and the perceived customization of the system. Furthermore, an almost significant (p = 0.069) association was noted for the variable “intention for future reuse,” though the p-value did not reach the conventional threshold for statistical significance (Table 4).

**Table 3:** Personalization of advice with Chi-Square Test

			Personalization of advice		
Age groups	N	%	Mean	SD	P value
18–24	22	19.64%	2.18	0.64	0.047
25–34	21	18.75%	2.25	1.00	
35–44	25	22.32%	2.20	0.83	
45–54	28	25.00%	2.17	0.83	
55 +	16	14.29%	2.55	0.93

**Table 4:** Customization of the system and future reuse with mann–whitney U test

	Gender	N	Mean	SD	P value
Customization of the system	Male	35	2.51	0.66	0.037
	Female	77	2.22	0.64	
	Total	112			
Future reuse	Male	35	2.86	0.73	0.069
	Female	77	2.57	0.83	
	Total	112			

**Fig. 9:**
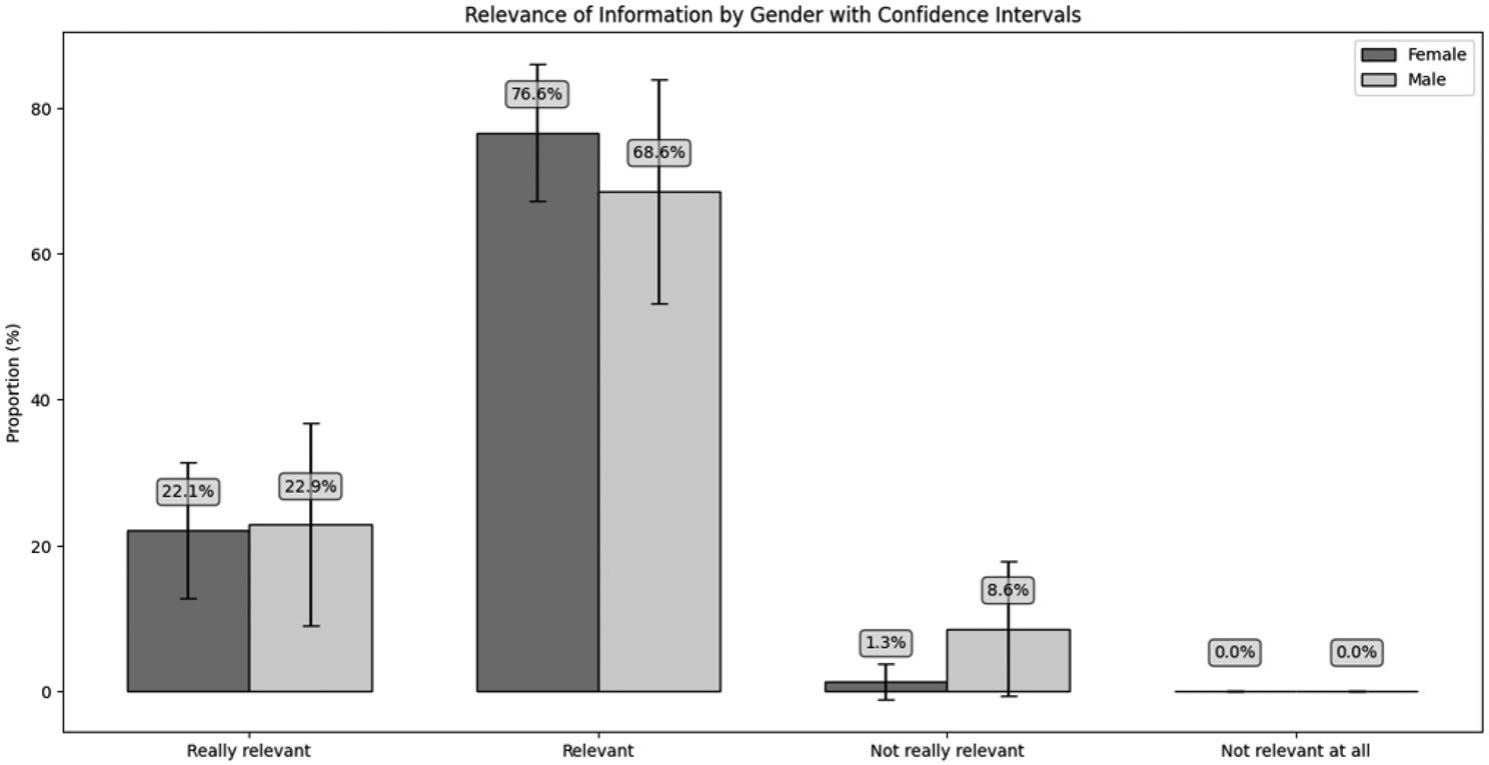
Tester perception of the relevance of information provided by the application on cervical cancer screening

It is also notable that both male and female testers reported that the information provided by the system regarding cervical cancer screening was highly relevant and informative (Fig. 9). From male testers, 22.9% and from female testers, 22.1% found the information really relevant, while 68.6% of male testers and 76.6% of female testers found it relevant. Only 8.6% of the male group and 1.3% of the female group found the information not relevant, and nobody reported that it was not relevant at all.

### Results of the system’s response evaluation by medical doctors

For each evaluation dimension (Accuracy, Clarity, and Usefulness), we computed the mean score and standard deviation across all questions. The mean scores were high for both MD1 and MD2 (all > 3.5), and the standard deviations were low, particularly in the Clarity and Usefulness dimensions, suggesting a concentrated response distribution. Due to the extremely low variability in the ratings, where the standard deviation was minimal and, in some cases, zero, it was not possible to compute a reliable correlation coefficient. Specifically, the Intraclass Correlation Coefficient (ICC) could not be calculated. Consequently, we employed the Wilcoxon Signed Ranks Test, which is designed to assess whether the median difference between two paired sets of observations is statistically indistinguishable from zero. For Accuracy, although the p-value was 0.059—very close to the conventional significance threshold of 0.05—it did not reach statistical significance. This indicates that there is insufficient evidence to conclude that the Accuracy ratings differ between MD1 and MD2. Similarly, in the case of Clarity (p = 0.063) and Usefulness (p = 0.317), the differences were not statistically significant. The lack of statistically significant differences in Accuracy, Clarity, and Usefulness indicates that the responses evaluated by MD1 and MD2 are essentially equivalent. This alignment supports the conclusion that the evaluations are coherent, with both sets of answers yielding very similar assessments and demonstrating consistency in the system’s responses across independent evaluators (Table 5).

**Table 5:** Descriptive statistics and wilcoxon signed-rank test results for independent evaluations on accuracy, clarity, and usefulness

	Mean	SD	Wilcoxon Signed Ranks Test P-value
Accuracy MD_1	3.83	0.38	0.059
Accuracy MD_2	3.57	0.50	
Clarity MD_1	3.80	0.55	0.063
Clarity MD_2	4.00	0	
Usefulness MD_1	3.97	0.18	0.317
Usefulness MD_2	4	0	

## Discussion

Our hypothesis posited that a custom GPT model enhances the accessibility, comprehensibility, and adherence to cervical cancer guidelines, thereby improving patient education and assisting patients in accessing specific information. According to our results, both male and female participants found the information provided by the system to be relevant and informative. Female participants particularly valued the guidance on screening schedules and the importance of early detection. Male participants noted the usefulness of the information provided on HPV vaccination and its role in preventing HPV-related cancers, a key area of concern given that males are often less engaged in HPV prevention efforts. Participants highlighted the user-friendly interface and the empathetic responses generated by the system, which made the experience more engaging. This feedback is crucial, as it demonstrates the application’s potential in supporting public health efforts by encouraging individuals to engage in preventive measures such as screening and HPV vaccination. We could only identify a statistically significant association between age groups and the perceived personalization of the responses. We also observed a significant association between gender and the perceived customization of the system. Furthermore, an association was noted for the variable “intention for future reuse,” though the p-value did not reach the conventional threshold for statistical significance. All the other results for other variables were not significant. Another notable finding was the higher level of engagement reported by younger participants. They described the interface as more interactive and user-friendly compared for users over 40 years old. Although the correlation between age and system interactivity level was not statistically significant, it is essential to explore tools and design strategies that can enhance the accessibility and interactivity of custom GPT systems. Such observations underscore the importance of considering age-related preferences in the design and optimization of digital health applications. Our current sample primarily comprises participants aged 25–54 in accordance with the most recent World Health Organization (WHO) guidance on cervical cancer prevention and control. Routine screening is prioritized for women aged 30–49 and remains applicable up to the age of 65. Although screening can still be important for women over 65, we believe that developing a digital patient education tool about cervical cancer screening is most critical for women aged 25–49, the group that constituted the majority of our participants. Moreover, younger individuals are generally more attracted to digital tools, which was a key reason for selecting cervical cancer screening as the focus for our custom GPT model. In contrast, other screening tests, such as mammography and colorectal screening, typically commence later in a patient’s life and are more relevant for older populations. It is also important to note that the study was conducted in Hungary. The generalizability of the findings to other populations and healthcare systems should be further examined in future studies.

To the best of our knowledge, while several AI-based tools have been developed for cervical cancer screening, the majority of these focus on image analysis for diagnostic support [51–54]. In contrast, our solution is an LLM-based patient education tool designed to provide information about cervical cancer and enhance access to publicly available knowledge. We believed that this approach would address a critical gap in healthcare access, as many individuals miss out on potentially life-saving screening tests due to insufficient information.

In comparison to other health chatbot tools, it is primarily concluded that AI chatbots have proven effective in promoting health behavior changes across large and diverse populations, just as shown in our results. Studies have shown that LLMs are capable of generating accurate, readable, and contextually relevant responses to patient queries, making them valuable tools for improving health literacy and supporting doctor-patient communication. Scoping reviews highlight their use in creating patient education materials, simplifying complex medical information, and enhancing accessibility [55–57]. Specifically in the context of cervical cancer screening, research has demonstrated that LLMs generally provide responses aligned with established clinical guidelines. They show potential in supporting patient understanding of screening procedures, risks, and follow-up care [58, 59]. However, future research should focus on conducting rigorous randomized controlled trials to draw definitive conclusions [60, 61]. The studies generally showed positive or mixed results regarding the effectiveness, usability, and user satisfaction of the conversational agents examined, although qualitative user feedback was more varied, just as in our results. Many of the studies had limitations in quality, highlighting the need for better study design and reporting to more accurately assess the agents’ usefulness in healthcare and pinpoint areas for improvement. Future research should also focus on evaluating the cost-effectiveness, privacy, and security of these agents [62]. As the clinical use of LLMs grows, ethical, regulatory, and patient safety concerns will become increasingly important [63].

Despite the advantages of creating a custom LLM for a specific healthcare area, several limitations must be considered. While it demonstrates potential in promoting awareness and encouraging individuals to seek information about cervical cancer screening and HPV vaccination, its conversational capabilities are subject to the inherent limitations of current language model technology, specifically in this case, the GPT-4 model. This model relies on advanced statistical methods to generate responses based on patterns in data rather than true comprehension or reasoning. Consequently, its ability to address user inquiries with complete accuracy and nuance may be limited, highlighting the importance of complementing AI-generated information with professional medical advice. Prompt engineering is integral to guiding large language models in specialized domains such as healthcare. However, managing ambiguous user queries remains a key challenge; uncertain or incomplete inputs can lead to imprecise responses, underscoring the need for dynamic query refinement and interactive clarifications. Moreover, even when a system relies exclusively on prompt engineering and is not further fine-tuned, it inherits biases or limitations embedded in the model’s pretraining corpus; these cannot be fully mitigated by prompt design alone. Consequently, routine data audits, broader data sources, and structured bias detection strategies are essential, particularly in clinical contexts where equity and reliability are paramount. An iterative process of prompt refinement, enhanced by user feedback loops, ensures alignment with evolving medical guidelines and real-world needs.

AI hallucination refers to the phenomenon wherein artificial intelligence systems, particularly LLMs, generate outputs that are factually inaccurate, logically inconsistent, or fabricated. This phenomenon arises from the probabilistic nature of LLMs, which predict responses based on statistical patterns within training data rather than genuine comprehension. This issue presents significant challenges, particularly in high-stakes domains such as healthcare and scientific research, where precision and reliability are paramount. However, its performance can be enhanced through various techniques, although this requires extensive testing and continuous refinement. There are many options and approaches to address these challenges [64–66]. In our paper, we employed HITL evaluation and two independent medical doctors evaluations. Further strategies for bias detection and mitigation can be explored in future studies, including gender, cultural, and socioeconomic biases, which may affect both the accuracy and fairness of their outputs. Informed consent is another critical ethical consideration. Users must be fully informed about how their data will be collected, stored, and used, as well as the limitations of AI-generated recommendations. Clear and accessible consent forms should outline users’ rights, including the option to withdraw their data at any time. Additionally, users should be made aware that AI systems are designed to assist rather than replace healthcare professionals, emphasizing the importance of consulting qualified experts for personalized medical advice.

The user experience was evaluated using a questionnaire tailored specifically for this study. In this preliminary study, our primary goal was to gather early insights into user needs and directions for system refinement, to best serve this goal, we employed a brief, customized questionnaire. Although this instrument captured key usability metrics, its scope was limited. Future research would benefit from integrating standardized instruments such as the System Usability Scale (SUS) or the Post-Study System Usability Questionnaire (PSSUQ) to gather immediate post-task usability feedback. These additional measures may offer a more comprehensive assessment of the system’s performance and user satisfaction.

The widespread adoption of custom GPTs in healthcare systems also raises new challenges, including ethical considerations, security concerns, and the need for transparent governance in their application. The development of such a model requires a significant initial investment of time, expertise, and resources, including processing official medical guidelines and even collaboration with healthcare professionals to ensure accuracy and clinical relevance. Ensuring the model’s ability to handle the complexities of medical information across diverse patient profiles is another challenge. Ethical concerns arise around data privacy, security, and the potential for misuse, particularly if patients overly rely on automated systems rather than seeking direct care from healthcare professionals. Ensuring robust data privacy and security measures is essential when developing AI-driven systems, particularly in healthcare. A potential solution is the implementation of data minimization techniques, which limit the collection and processing of personal information to only what is strictly necessary. In this study, the system was designed to respond to general questions regarding cervical cancer screening, tailored to the tester’s individual needs, while ensuring that no sensitive or identifiable data was collected or used. This approach enhances privacy by reducing the risk of unauthorized access and ensures compliance with data protection regulations.

## Conclusion

In conclusion, we successfully developed a custom GPT model focused on cervical cancer screening by processing medical guidelines and utilizing prompt engineering techniques, the OpenAI GPT-4 model, and Python programming language. The system was integrated into the Streamlit framework, allowing for seamless deployment and beta testing with 115 participants during a three-week period. A total of 112 testers filled out the user experience questionnaire and provided feedback on various aspects of the system. Additionally, two independent medical doctors evaluated the answers for the 30 most frequently asked questions.

This study highlights the potential of AI-driven systems to enhance cervical cancer screening coverage and mitigate healthcare resource shortages, particularly in resource-constrained settings. By delivering interactive, personalized health information, tailored to individual needs, such systems can improve public awareness and encourage greater participation in preventive healthcare. The effectiveness of such an AI-driven approach lies in its ability to simplify complex medical information, making it both accessible and actionable for a diverse population. Moreover, the system’s capability to generate empathetic and motivational responses—simulating a conversation with a supportive and knowledgeable entity—fosters user engagement and trust. These findings underscore the broader applicability of AI-powered conversational models in advancing health communication and promoting evidence-based preventive healthcare strategies.

According to our results and a comparison with findings in the literature, we concluded that while AI will never fully substitute healthcare professionals, it can serve as a valuable complement, enhancing the efficiency of healthcare delivery. AI systems can streamline workflows, assist in routine tasks, and provide patients with quicker access to information, ultimately improving the overall healthcare experience. By alleviating some of the administrative and informational burdens on healthcare providers, AI enables professionals to focus more on critical decision-making and personalized patient care.

Long-term user engagement and satisfaction are critical for the effectiveness of digital health tools. Future work will investigate strategies such as enhanced personalization, user-centered design improvements, and the integration of interactive educational features to promote sustained use and increase the likelihood of user recommendations. Future iterations and testing will also focus on broadening the system’s conversational scope to include critical preventive screening examinations, such as those for breast and colorectal cancer.

## Electronic supplementary material

Below is the link to the electronic supplementary material.

Supplementary Material 1

Supplementary Material 2

Supplementary Material 3

## Data Availability

All data generated or analysed during this study are included in this published article and its supplementary information files. [User_experience_questionaire_data] [Webapplication_data].

## Abbreviations

API: Application Programming Interface
AI: Artificial Intelligence
GPT: Generative Pre-Trained Transformer
LLM: Large Language Model
NLP: Natural Language Processing
HITL: Human-in-the-Loop
HPV: Human Papillomavirus
LMIC: Low- and middle-income countries
UI: User Interface
PSSUQ: Post-Study System Usability Questionnaire
UEQ: User Experience Questionnaire
CUQ: Chatbot Usability Questionnaire
SUS: System Usability Scale

## Declarations

### Testing the custom GPT model

A question flow (Table 1 and Supplementary 1) was applied to beta test the custom GPT model. An informed consent was deemed unnecessary for the testing as it only utilized anonymized, non-sensitive data that adhered to the General Data Protection Regulation (GDPR), including Regulation (EU) 2016/679 Article 9. It is only aimed to customize the custom GPT model’s answers for individual needs. Participants voluntarily engaged in the process, indicating their agreement to use the custom GPT model. An English version of the survey is provided as a supplementary file.

### Measuring the user experience of the custom GPT

Participants who tested the custom GPT model had the chance to voluntarily and anonymously complete a user experience questionnaire. Informed consent was obtained here, and respondents aged 18 and above were included. The user experience questionnaire model did not collect any personally identifiable healthcare data as well.

### Relevant guidelines and regulations

- World Helath Organization Ethics and governance of artificial intelligence for health: Guidance on large multi-modal models

- Hungarian Act CLIV of 1997, §157 “Medical research conducted on humans”

- GDPR, including Regulation (EU) 2016/679 Article 9.

- World Medical Association’s Declaration of Helsinki

## References

[1] De Angelis L, et al. ChatGPT and the Rise of Large Language Models: The New AI-driven Infodemic Threat in Public Health. Front. Public Health. 2023;11:1166120. 10.3389/fpubh.2023.1166120 PMC10166793 37181697

[2] Bhargava DC, et al. ChatGPT in medical research: challenging time ahead. Med Leg J. 2023;91:223–25. 37802491 10.1177/00258172231184548

[3] Li J, et al. ChatGPT in healthcare: a taxonomy and systematic review. Comput Methods Programs Biomed. 2024;245:108013. 10.1016/j.cmpb.2024.108013 38262126

[4] Liu CL, Ho CT, Wu TC. Custom GPTs enhancing performance and evidence compared with GPT-3.5, GPT-4, and GPT-4o? A study on the emergency medicine specialist examination. Healthcare (Basel). 2024;12(17). 10.3390/healthcare12171726 PMC11394718 39273750

[5] Fisher AD, Fisher G. Evaluating performance of custom GPT in anesthesia practice. J Clin Anesth. 2023;93:111371. 38154443 10.1016/j.jclinane.2023.111371

[6] Sathe TS, et al. How I GPT It: development of Custom Artificial Intelligence (AI) chatbots for surgical education. J Surg Educ. 2024;81:772–75. 38627117 10.1016/j.jsurg.2024.03.004

[7] Darkhabani M, et al. ChatGPT and autoimmunity - A new weapon in the battlefield of knowledge. Autoimmun Rev. 2023;22:103360. 37211242 10.1016/j.autrev.2023.103360

[8] Sun H, et al. An AI dietitian for type 2 diabetes mellitus management based on large language and image recognition models: preclinical concept validation study. J Med Internet Res. 2023;25:e51300. 37943581 10.2196/51300 PMC10667983

[9] Spallek S, et al. Can we use ChatGPT for mental health and substance use education? examining its quality and potential harms. JMIR Med Educ. 2023;9:e51243. 38032714 10.2196/51243 PMC10722374

[10] Haver HL, et al. Appropriateness of breast cancer prevention and screening recommendations provided by ChatGPT. Radiology. 2023;307:e230424. 37014239 10.1148/radiol.230424

[11] Zheng Y, et al. Enhancing diabetes self-management and education: a critical analysis of ChatGPT’s role. Ann Biomed Eng. 2023. 10.1007/s10439-023-03317-8 37553556

[12] Cascella M, et al. Evaluating the feasibility of chatgpt in healthcare: an analysis of multiple clinical and research scenarios. J Med Syst. 2023;47:33. 36869927 10.1007/s10916-023-01925-4 PMC9985086

[13] Rao A, et al., Evaluating ChatGPT as an Adjunct for Radiologic Decision-Making. 2023, Cold Spring Harbor Laboratory.

[14] Mondal H, et al. ChatGPT in answering queries related to lifestyle-related diseases and disorders. Cureus. 2023;15:e48296. 38058315 10.7759/cureus.48296 PMC10696911

[15] Meskó B. Prompt engineering as an important emerging skill for medical professionals: tutorial. J Med Internet Res. 2023;25:e50638. 37792434 10.2196/50638 PMC10585440

[16] Zaghir J, et al. Prompt engineering paradigms for medical applications: scoping review. J Med Internet Res. 2024;26:e60501. 39255030 10.2196/60501 PMC11422740

[17] Chen JS, Granet DB. Prompt engineering: helping ChatGPT respond better to patients and parents. J Pediatr Ophthalmol Strabismus. 2024;61(2):148–49. 38529749 10.3928/01913913-20240124-02

[18] Giray L. Prompt Engineering with ChatGPT: a guide for academic writers. Ann Biomed Eng. 2023;51(12):2629–33. 37284994 10.1007/s10439-023-03272-4

[19] Li Y. A Practical Survey on Zero-shot prompt design for in-context learning. 2023.

[20] Larbi D, Denecke K, Gabarron E. Usability Testing of a social media chatbot for increasing physical activity behavior. Journal of Personalized Medicine. 2022;12:828. 35629252 10.3390/jpm12050828 PMC9144074

[21] Cheah WH, et al. Mobile technology in medicine: development and validation of an adapted system usability scale (sus) questionnaire and modified technology acceptance model (TAM) to evaluate user experience and acceptability of a mobile application in MRI safety screening. Indian J Radiol Imaging. 2023;33:36–45. 36855734 10.1055/s-0042-1758198 PMC9968523

[22] Zhu D, et al. User Interface (UI) design and user experience questionnaire (UEQ) evaluation of a to-do list mobile application to support day-to-day life of older adults. Healthcare (Basel). 2022;10(10). 10.3390/healthcare10102068 PMC9602058 36292518

[23] Zhou L, et al. The mHealth app usability questionnaire (MAUQ): development and validation study. JMIR Mhealth Uhealth. 2019;7:e11500. 30973342 10.2196/11500 PMC6482399

[24] Holderried F, et al. A generative pretrained transformer (GPT)-powered chatbot as a simulated patient to practice history taking: prospective, mixed methods study. JMIR Med Educ. 2024;10:e53961. 38227363 10.2196/53961 PMC10828948

[25] Miguel Cruz A, et al. Acceptance, adoption, and usability of information and communication technologies for people living with dementia and their care partners: a systematic review. Disabil Rehabil Assist Technol. 2023;18:443–57. 33378627 10.1080/17483107.2020.1864671

[26] Fontham ETH, et al. Cervical cancer screening for individuals at average risk: 2020 guideline update from the american cancer society. CA Cancer J Clin. 2020;70:321–46. 32729638 10.3322/caac.21628

[27] WHO Guidelines approved by the guidelines review committee, in WHO guideline for screening and treatment of cervical pre-cancer lesions for cervical cancer prevention: use of mRNA tests for human papillomavirus (HPV) World Health Organization World Health Organization 2021 Geneva. 35044737

[28] Sultanov M, et al. Investigating feasibility of 2021 WHO protocol for cervical cancer screening in underscreened populations: pREvention and SCReening innovation project toward elimination of cervical cancer (PRESCRIP-TEC). BMC Public Health. 2022;22(1). 10.1186/s12889-022-13488-z PMC9284962 35840949

[29] WHO Guidelines approved by the guidelines review committee, in WHO guideline for screening and treatment of cervical pre-cancer lesions for cervical cancer prevention: use of dual-stain cytology to triage women after a positive test for human papillomavirus (HPV) World Health Organization World Health Organization 2024 Geneva. 38976622

[30] Gavinski K, DiNardo D. Cervical Cancer screening. Med Clin North Am. 2023;107(2):259–69. 36759096 10.1016/j.mcna.2022.10.006

[31] Bhatla N, Singhal S. *Primary HPV screening for cervical cancer.* best pract res clin obstet gynaecol. 2020;65:98–108. 10.1016/j.bpobgyn.2020.02.008 32291178

[32] Voelker RA. Cervical cancer screening. Jama. 2023;330(20):2030. 37889510 10.1001/jama.2023.21987

[33] Popalis ML, et al. Improving cervical cancer screening rates: a scoping review of resources and interventions. Cancer Causes Control. 2022;33:1325–33. 35980511 10.1007/s10552-022-01618-2 PMC10124066

[34] Varatharajan S, et al. Cervical cancer in Malaysia: can we improve our screening and preventive practice? BMC Public Health. 2012;12:A17.

[35] Vidrine JI, et al. Enhancing long-term smoking abstinence among individuals with a history of cervical intraepithelial neoplasia or cervical cancer (Project ACCESS): protocol for a randomized clinical trial. BMC Public Health. 2023;23(1). 10.1186/s12889-023-16189-3 PMC10318835 37403057

[36] Nishimura Y, et al. Mailing human papillomavirus self-sampling kits to women under-screened for cervical cancer improved detection in cervical cancer screening in a general population study in Japan. BMC Public Health. 2023;23(1). 10.1186/s12889-023-15402-7 PMC10008572 36906527

[37] Tao L, et al. Prevalence and risk factors for cervical neoplasia: a cervical cancer screening program in Beijing. BMC Public Health. 2014;14:1185. 25410572 10.1186/1471-2458-14-1185 PMC4256817

[38] Hong Y, et al. HPV and cervical cancer related knowledge, awareness and testing behaviors in a community sample of female sex workers in China. BMC Public Health. 2013;13:696. 23898889 10.1186/1471-2458-13-696 PMC3733604

[39] Scott Duncan T, et al. Empowered patients and informal care-givers as partners?-a survey study of healthcare professionals’ perceptions. BMC Health Serv Res. 2023;23:404. 37101266 10.1186/s12913-023-09386-8 PMC10131407

[40] Girasek E, et al. E-páciensek Magyarországon: digitális egészséggel kapcsolatos ismeretek, szokások egy országos reprezentatív felmérés tükrében. Orvosi Hetilap 2022;163:1159–65. 35895447 10.1556/650.2022.32512

[41] Chen P, et al. The acceptability and effectiveness of artificial intelligence-based chatbot for hypertensive patients in community: protocol for a mixed-methods study. BMC Public Health. 2024;24(1). 10.1186/s12889-024-19667-4 PMC11337738 39169305

[42] Péter Dr. Bôsze, G.p.d. Hernádi Zoltán Dr, Károly Dr Pap. Ungár László DR, *A méhnyakrák szûrésének szempontjai: hazai irányelvek*. Nőgyógyászati Onkológia. 2009;14:11–17.

[43] Fisher S, Rosella LC. Priorities for successful use of artificial intelligence by public health organizations: a literature review. BMC Public Health. 2022;22(1). 10.1186/s12889-022-14422-z PMC9682716 36419010

[44] Morgenstern JD, et al. “AI’s gonna have an impact on everything in society, so it has to have an impact on public health”: a fundamental qualitative descriptive study of the implications of artificial intelligence for public health. BMC Public Health. 2021;21(1). 10.1186/s12889-020-10030-x PMC7787411 33407254

[45] Gynecology PCOOA. The professional protocol of the hungarian ministry of health - cervical cancer. 2005.

[46] Green J, et al. Concomitant chemotherapy and radiation therapy for cancer of the uterine cervix. Cochrane Database Syst Rev 2005;2005:Cd002225. 16034873 10.1002/14651858.CD002225.pub2 PMC10634661

[47] Shueng PW, et al. Neoadjuvant chemotherapy followed by radiotherapy should not be a standard approach for locally advanced cervical cancer. Int J Radiat Oncol Biol Phys. 1998;40:889–96. 9531375 10.1016/s0360-3016(97)00906-1

[48] Einhorn N, et al. A systematic overview of radiation therapy effects in cervical cancer (cervix uteri). Acta Oncol 2003;42:546–56. 14596512 10.1080/02841860310014660

[49] Center HNPHAP, Cervical Cancer Screening

[50] OpenAI, OpenAI API Reference. 2024.

[51] Viñals R, et al. artificial intelligence-based cervical cancer screening on images taken during visual inspection with acetic acid: a systematic review. Diagnostics (Basel). 2023;13(5). 10.3390/diagnostics13050836 PMC10001377 36899979

[52] Kanavati F, et al. A Deep learning model for cervical cancer screening on liquid-based cytology specimens in whole slide images. Cancers (Basel). 2022;14(5). 10.3390/cancers14051159 PMC8909106 35267466

[53] Mathivanan SK, et al. Enhancing cervical cancer detection and robust classification through a fusion of deep learning models. Sci Rep. 2024;14:10812. 38734714 10.1038/s41598-024-61063-w PMC11088661

[54] Hou X, et al. Artificial intelligence in cervical cancer screening and diagnosis. Front Oncol. 2022;12:851367. 35359358 10.3389/fonc.2022.851367 PMC8963491

[55] AlSammarraie A, Househ M.The use of large language models in generating patient education materials: a scoping review. Acta Inform Med. 2025;33(1):4–10. 40223858 10.5455/aim.2024.33.4-10 PMC11986337

[56] Aydin S, et al. Large language models in patient education: a scoping review of applications in medicine. Front Med (Lausanne). 2024;11:1477898. 39534227 10.3389/fmed.2024.1477898 PMC11554522

[57] Lin C, Kuo CF. Roles and potential of large language models in healthcare: a comprehensive review. Biomed J. 2025;100868.10.1016/j.bj.2025.10086840311872

[58] Reicher L, et al. Exploring the role of artificial intelligence, large language models: comparing patient-focused information and clinical decision support capabilities to the gynecologic oncology guidelines. Int J Gynaecol Obstet. 2025;168:419–27. 39161265 10.1002/ijgo.15869 PMC11726133

[59] Kuerbanjiang W, et al. Performance evaluation of large language models in cervical cancer management based on a standardized questionnaire: comparative study. J Med Internet Res. 2025;27:e63626. 39908540 10.2196/63626 PMC11840365

[60] Aggarwal A, et al. Artificial intelligence-based chatbots for promoting health behavioral changes: systematic review. J Med Internet Res. 2023;25:e40789. 36826990 10.2196/40789 PMC10007007

[61] Xu L, et al. Chatbot for health care and oncology applications using artificial intelligence and machine learning: systematic review. JMIR Cancer. 2021;7:e27850. 34847056 10.2196/27850 PMC8669585

[62] Milne-Ives M, et al. The effectiveness of artificial intelligence conversational agents in health care: systematic review. J Med Internet Res. 2020;22:e20346. 33090118 10.2196/20346 PMC7644372

[63] Huo B, et al. Large language models for chatbot health advice studies: a systematic review. JAMA Netw Open. 2025;8:e2457879. 39903463 10.1001/jamanetworkopen.2024.57879 PMC11795331

[64] Chen X, et al. EyeGPT for patient inquiries and medical education: development and validation of an ophthalmology large language model. J Med Internet Res. 2024;26:e60063. 39661433 10.2196/60063 PMC11669878

[65] Wilhelm TI, Roos J, Kaczmarczyk R. Large language models for therapy recommendations across 3 clinical specialties: comparative study. J Med Internet Res. 2023;25:e49324. 37902826 10.2196/49324 PMC10644179

[66] Zhu L, et al. Testing and validation of a custom retrained large language model for the supportive care of hn patients with external knowledge base. Cancers (Basel). 2024;16(13). 10.3390/cancers16132311 PMC11240646 39001375

