# Supplementary material for: Exploring the possibilities and limitations of customized large language model to support and improve cervical cancer screening
Source: BMC Med Inform Decis Mak. 2025 Jul 1;25:242. doi: 10.1186/s12911-025-03088-3 (PMC12220158; doi:10.1186/s12911-025-03088-3)
Supplement: Supplementary file 1 — Supplementary Material 1 [file 12911_2025_3088_MOESM1_ESM.docx]

**Initial survey questions**

**Question flow 1.: Female under 25 years old (over 18 years old)**

1. Age, gender
2. Are you sexually active?
3. Do you currently use any protection, like condoms, to reduce the risk of sexually transmitted infections (STIs)?
4. Have you received the HPV vaccine?
5. Do you have any lifestyle factors or habits, such as smoking or a weakened immune system, that might affect your risk of cervical cancer?

**Question flow 2.: Female over 25 years old**

1. Age, gender
2. Are you sexually active?
3. Do you currently use any protection, like condoms, to reduce the risk of sexually transmitted infections (STIs)?
4. Have you received the HPV vaccine?
5. Do you have any lifestyle factors or habits, such as smoking or a weakened immune system, that might affect your risk of cervical cancer?
6. Has anyone in your family ever had cervical cancer or other gynaecological diseases?
7. Have you ever had a cervical cancer screening related examination, such as a Pap test or an HPV test?
8. Have you screened in within the last 3 years?
9. Have you experienced any abnormal symptoms recently, such as unusual bleeding or pelvic pain?

**Question flow 3.: Male at any age (over 18 years old)**

1. Age, gender
2. Are you sexually active?
3. Have you received the HPV vaccine?

The above set of survey questions represents the initial queries employed by our customized GPT model to create a more personalized experience for the testers. Medical guidelines and recommendations related to cervical cancer, including HPV vaccination and prevention, were gathered to develop the question flow for our web application aimed to customize the system as much as possible for individual needs. The guidelines processed included those from the American Cancer Society and WHO for cervical cancer screening and treatment of pre-cancerous lesions, as well as relevant scientific publications. Additionally, two Hungarian guidelines—the Hungarian Ministry of Health’s Professional Protocol and recommendations from the Hungarian National Public Health and Pharmaceutical Center—were incorporated, as the system was tested in Hungary. Please see list of relevant references below. The short survey was developed specifically for this research.

1. Fontham, E.T.H., et al., Cervical cancer screening for individuals at average risk: 2020 guideline update from the American Cancer Society. CA Cancer J Clin, 2020. 70(5): p. 321-346.

2. WHO Guidelines Approved by the Guidelines Review Committee, in WHO guideline for screening and treatment of cervical pre-cancer lesions for cervical cancer prevention: Use of mRNA tests for human papillomavirus (HPV). 2021, World Health Organization Geneva.

3. Gynecology, P.C.o.O.a., The Professional Protocol of the Hungarian Ministry of Health - Cervical Cancer. 2005.

4. Green, J., et al., Concomitant chemotherapy and radiation therapy for cancer of the uterine cervix. Cochrane Database Syst Rev, 2005. 2005(3): p. Cd002225.

5. Shueng, P.W., et al., Neoadjuvant chemotherapy followed by radiotherapy should not be a standard approach for locally advanced cervical cancer. Int J Radiat Oncol Biol Phys, 1998. 40(4): p. 889-96.

6. Einhorn, N., et al., A systematic overview of radiation therapy effects in cervical cancer (cervix uteri). Acta Oncol, 2003. 42(5-6): p. 546-56.

7. Center, H.N.P.H.a.P., Cervical Cancer Screening
