# Supplementary material for: Exploring the possibilities and limitations of customized large language model to support and improve cervical cancer screening
Source: BMC Med Inform Decis Mak. 2025 Jul 1;25:242. doi: 10.1186/s12911-025-03088-3 (PMC12220158; doi:10.1186/s12911-025-03088-3)
Supplement: Supplementary file 3 — Supplementary Material 3 [file 12911_2025_3088_MOESM3_ESM.docx]

30 Frequently asked questions related to cervical cancer

1. Why should I undergo cervical screening?

2. How can I participate in cervical screening?

3. How is cervical screening performed?

4. Who should undergo cervical screening?

5. What is cervical screening?

6. When is the optimal time for cervical cancer screening?

7. What is cervical cancer?

8. What causes cervical cancer?

9. Is cervical cancer caused by a viral infection?

10. How can one become infected with HPV?

11. Does Human Papillomavirus cause abnormalities only in women?

12. Does Human Papillomavirus only cause cervical cancer?

13. Do women who have already been infected with HPV develop antibodies against the virus, thereby eliminating the need for vaccination?

14. Does cervical cancer affect only older women?

15. Can cervical cancer be prevented?

16. Are women who take hormonal contraceptives at an increased risk of developing cervical cancer?

17. Can genital warts lead to cancer?

18. Am I at risk if I have not been sexually active for a long time?

19. What are the risk factors for cervical cancer?

20. What exactly happens during the screening process?

21. What happens if the cytological report is not negative?

22. What are the precancerous conditions of the cervix?

23. Does cervical cancer have clear and noticeable symptoms?

24. Is vaccination against cervical cancer recommended exclusively for young females?

25. Is regular gynecological cancer screening no longer required after receiving the cervical cancer vaccine?

26. How does cervical cancer spread?

27. What is cervical dysplasia?

28. Can men contract HPV?

29. Why are regular follow-up examinations important after cervical cancer treatment?

30. How does cervical cancer affect pregnancy?
